# Functional Characterization and Antifungal Activity of Insect-Derived Chitinases Expressed in *Pichia pastoris*

**DOI:** 10.3390/polym18030402

**Published:** 2026-02-03

**Authors:** Katia Celina Santos Correa, Gabriel Henrique Ribeiro, Odair Correa Bueno, Luiz Alberto Colnago, Iran Malavazi, Dulce Helena Ferreira de Souza

**Affiliations:** 1 Department of Chemistry, Federal University of Sao Carlos, São Carlos 13565-905, SP, Brazil; katiacorrea12@gmail.com; 2 Embrapa Instrumentation, Brazilian Agricultural Research Corporation, São Carlos 13560-970, SP, Brazil; gabrielhenri10@hotmail.com (G.H.R.); luiz.colnago@embrapa.br (L.A.C.); 3 Center for the Study of Social Insects, Sao Paulo State University ‘Julio de Mesquita Filho’, Rio Claro 13506-900, SP, Brazil; odair.bueno@unesp.br; 4 Department of Genetics and Evolution, Federal University of São Carlos, São Carlos 13565-905, SP, Brazil; imalavazi@ufscar.br

**Keywords:** insect chitinases, antifungal activity, recombinant chitinases, biocatalysis, biotechnology applications, insect-derived enzymes

## Abstract

Chitinases catalyze the hydrolysis of β-1,4-glycosidic bonds in chitin, a structural biopolymer synthesized by numerous organisms. Although these enzymes have been widely investigated, studies focusing on insect-derived chitinases remain limited. In this study, three recombinant chitinases from the leaf-cutter ant *Atta sexdens* were cloned, expressed in *Pichia pastoris*, and biochemically characterized. The enzymes-AsChtII-C2B3 (one catalytic and three chitin-binding domains), AsChtII-C3C4 (two catalytic domains), and AsChtII-C5B1 (one catalytic and one binding domain), exhibited optimal activity at pH 4–5 and 50 °C using colloidal chitin as substrate. Chitinase activity on colloidal α-chitin was confirmed by ^1^H NMR (proton nuclear magnetic resonance) spectroscopy, revealing GlcNAc concentrations of 0.41, 0.48, and 0.56 mmol L^−1^ for AsChtII-C3C4, AsChtII-C2B3, and AsChtII-C5B1, respectively. Their antifungal activities were evaluated against the human pathogens *Candida albicans* and *Aspergillus fumigatus*, as well as the phytopathogen *Lasiodiplodia theobromae*. Distinct inhibition profiles were observed: AsChtII-C5B1 (150 µg/mL) showed the highest activity against *C. albicans* (87.6% inhibition), while AsChtII-C3C4 (25 µg/mL) was most effective against *A. fumigatus* (60% inhibition). Notably, only AsChtII-C2B3 inhibited *L. theobromae* growth, inducing severe hyphal deformations observed by scanning electron microscopy (SEM). These findings demonstrate that recombinant *A. sexdens* chitinases exhibit species-specific antifungal properties, underscoring their potential as biotechnological tools for medical and agricultural applications.

## 1. Introduction

Chitin is a natural polymer composed of N-acetylglucosamine (GlcNAc) units linked by β-(1→4) glycosidic bonds. It is the second most abundant biopolymer in nature and a major structural component of fungal cell walls, insect exoskeletons, and the peritrophic membrane of arthropods [1,2]. Chitin biosynthesis is catalyzed by chitin synthases (EC 2.4.1.16), which are membrane-associated glycosyltransferases responsible for the polymerization of UDP-N-acetylglucosamine into linear chitin chains [3].

Chitin turnover requires the coordinated activity of chitin synthases and chitinases (EC 3.2.1.14). Chitinases hydrolyze β-(1→4) glycosidic bonds in chitin polymers and oligomers, releasing N-acetylglucosamine and soluble chito-oligosaccharides [4,5]. These enzymes are widely distributed among organisms, including fungi, crustaceans, insects, plants, bacteria, viruses, and vertebrates, regardless of whether they synthesize chitin [6].

Chitinolytic enzymes have been studied for their potential technological applications. Products generated from chitin hydrolysis, particularly chito-oligosaccharides, have been investigated for use in pharmaceuticals, cosmetics, food supplements, agriculture, and biomaterials [7,8,9,10]. Because chitin is absent from plants and vertebrates, chitinases have also been evaluated as biological control agents against insects and fungal pathogens, with limited effects on non-target organisms [10,11].

In insects, chitinases belong mainly to glycosyl hydrolase family 18 (GH18) and have been identified in several insect orders, including Lepidoptera, Diptera, Hemiptera, Coleoptera, and Hymenoptera [12]. These enzymes are primarily expressed in epidermal tissues and are involved in physiological processes such as cuticle degradation during molting, growth, pupation, and regulation of the peritrophic membrane [13]. Insect chitinases differ in catalytic efficiency, substrate specificity, tissue distribution, developmental expression, and domain organization [14]. Based on sequence similarity and domain composition, they are classified into eleven groups, designated ChtI–ChtX and Chih [15].

ChtII chitinases are large multidomain enzymes containing multiple catalytic domains and chitin-binding modules (CBMs). Their catalytic mechanism depends on a conserved glutamic acid residue that participates in proton donation during cleavage of β-(1→4) glycosidic bonds [13]. Structural and functional analyses have shown that these enzymes possess distinct catalytic and binding features that contribute to efficient chitin degradation during insect molting and pupation [16,17,18,19].

Several insect chitinases have been explored for their potential use in pest control and combating fungal pathogens. For example, *Bombyx mori* chitinase has been evaluated as a biopesticide against the *Monochamus alternatus* beetle, demonstrating significant efficacy [20]. The chitinase of *Lacanobia oleracea* (tomato moth larva) showed an antagonistic effect against *Haemaphysalis longus*, reducing the thickness of the cuticle of this species [21]. Additionally, the chitinase from *Ostrinia furnacalis* has shown antifungal activity against plant pathogens such as *Fusarium graminearum* and *Botrytis cinerea* [22].

Previously, a recombinant chitinase derived from *Atta sexdens* showed larvicidal activity against *Spodoptera frugiperda* and antifungal activity against *Candida albicans*, *Aspergillus fumigatus*, and *Lasiodiplodia theobromae* [23,24]. Ants represent a substantial proportion of insect biomass and play important ecological roles. Species of the genera *Atta* and *Acromyrmex* are widely distributed in Neotropical regions and are recognized as significant herbivorous insects in agricultural systems [25,26].

The present study describes the production of three recombinant chitinases from *Atta sexdens* containing different combinations of catalytic domains and chitin-binding modules. The antifungal activity of these enzymes was evaluated against human pathogenic and phytopathogenic fungi to assess the relationship between domain architecture and biological activity.

## 2. Materials and Methods

### 2.1. Total RNA Extraction and cDNA Synthesis

Pupae of *A. sexdens* were collected from a nest maintained in the laboratory at the Center of Studies on Social Insects (UNESP, Rio Claro, Brazil). Total RNA was extracted from the pupae of *A. sexdens* using a protocol modified of the TRIzol^®^ manufacturer’s instructions, further adapted for the single-step RNA extraction methodology [27]. The RNA preparation was subjected to reverse transcription using the SuperScript^®^ VILO Master Mix (Thermo Fisher Scientific, Waltham, MA, USA) according to the manufacturer’s instructions. The resulting cDNA was utilized as the DNA template in a polymerase chain reaction (PCR) to amplify three nucleotide sequences called here AsChtII-C2B3, AsChtII-C3C4, and AsChtII-C5B1.

### 2.2. Cloning of Chitinase DNAs from A. sexdens

Since the genome of *Atta sexdens* has not yet been sequenced, specific oligonucleotides were designed and synthesized based on the coding sequence of a chitinase gene from the related leaf-cutting ant *Acromyrmex echinatior* (NCBI Reference Sequence: XM_011067337.1), available in the NCBI database (http://www.ncbi.nlm.nih.gov/ accessed on 15 January 2021).

To obtain the target DNAs, a two-step PCR strategy was employed. In the first step, primers lacking restriction sites were designed to amplify regions flanking the desired sequence, thereby enhancing specificity and efficiency. This approach yielded higher concentrations of template DNA and reduced the risk of nonspecific amplification by minimizing the template size. In the second step, the resulting amplicons were used as templates for PCR with primers containing restriction enzyme recognition sites. Amplification was performed using Phusion™ High-Fidelity DNA Polymerase (Thermo Fisher Scientific), according to the manufacturer’s instructions. The sequences of all primers used in this study are provided in Table S1 (Supplementary Materials).

Each of the three amplified DNA molecules was individually cloned into the CloneJET plasmid (PCR Cloning Kit vector system, Thermo Scientific, Waltham, MA, USA), following the manufacturer’s protocol. The resulting constructs were then transformed into *Escherichia coli* DH5α competent cells.

Transformed cells were plated on LB agar medium supplemented with ampicillin (100 μg/mL) and incubated at 37 °C for 16 h. Individual colonies were subsequently grown in LB medium containing ampicillin (100 μg/mL). Plasmid DNA from each clone was extracted using the Fast-n-Easy Plasmid Mini-Prep Kit (Cellco Biotec do Brasil Ltda, Sao Carlos, Brazil). For cloning efficiency, restriction analysis was performed using *EcoRI* and *NotI* for AsChtII-C2B3 and AsChtII-C5B1, and *SfiI* and *KpnI* for AsChtII-C3C4 followed by agarose gel electrophoresis. DNA was quantified using a BioSpec-nano spectrophotometer (Shimadzu Corporation, Kyoto, Japan) and sequenced using the ABI 3730 DNA Analyzer, a 48-capillary system (Life Technologies and Applied Biosystems, Carlsbad, USA). For subcloning into the pPICZαA vector (Invitrogen/Thermo Fisher, Waltham, MA, USA), the pJET clones of each construct were digested with the appropriate restriction enzymes and ligated into pPICZαA previously digested with the same enzymes.

The clones pPICZαA-AsChtII-C3C4 and pPICZαA-AsChtII-C5B1 were linearized with the *Sac*I enzyme (Thermo Scientific) while the clone pPICZαA-AsChtII-C2B3 was linearized with *Mss*I (PmeI) enzyme. All the DNAs were then used to transform the *P. pastoris* KM71H strain (Invitrogen) via electroporation. The transformed cells were seeded onto plates containing YPDS agar culture medium (yeast extract 1%, peptone 2%, dextrose 2%, sorbitol 1.0 M, agar 2%) supplemented with 100 µg/mL of zeocin. Grown colonies were seeded in the same culture medium in the presence of 500 µg/mL of zeocin. Each grown colony had its genomic DNA extracted and used as a template molecule in PCR, to verify DNA amplification corresponding to the desired DNA.

### 2.3. Protein Expression and Purification

One positive clone from each construct was grown in 10 mL of BMGY medium (1% yeast extract, 1% glycerol, 2% peptone, 0.2% biotin, 1.34% yeast nitrogen, 100 mM potassium phosphate, pH 6.0), supplemented with Zeocin (100 μg/mL) for 24 h at 29 °C and 250 rpm. The culture medium was then diluted into 500 mL of BMGY and incubated at the same conditions for additional 24 h. The solution was centrifuged at 1500× *g* for 5 min at 4 °C, and the pellet was resuspended in 100 mL of BMMY medium (1% yeast extract, 0.5% methanol, 2% peptone, 0.2% biotin, 1.34% yeast nitrogen, and 100 mM potassium phosphate, pH 6.0). The culture was maintained at 29 °C and 250 rpm for a period of 144 h, with methanol added every 24 h to final concentration of 1% (*v*/*v*). Subsequently, the culture medium was centrifuged at 3800× *g* for 8 min at 4 °C to remove cells. The resulting supernatant was subjected to ammonium sulfate precipitation (80% *w*/*v*) at room temperature, followed by centrifugation at 10,300× *g* for 45 min at 4 °C.

The pellet was resuspended in 5 mL of buffer A (50 mM sodium phosphate pH 5.9) and the solution was dialyzed using SnakeSkin (3.5 K MWCO) against buffer A at 4 °C for 3 h with three buffer changes. The second purification step was performed by affinity chromatography using a 2 mL Ni-NTA column (Qiagen, Hilden, Germany). The column was equilibrated with 6 mL of buffer A, and protein elution was carried out with 5 mL of buffer A containing 500 mM imidazole. The eluted protein was dialyzed against buffer A to remove the imidazole. The third purification step was performed using a size exclusion chromatography column, Superdex™ 75, with buffer A supplemented with 150 mM NaCl. The proteins were eluted at a flow rate of 0.5 mL/min. Protein concentration was determined by the Bradford method, employing bovine serum albumin (BSA) as the standard [28], and the purification process was analyzed on a 12% SDS-PAGE polyacrylamide gel.

### 2.4. Enzyme Assays

To assess enzymatic activity, 5% (*w*/*v*) colloidal chitin was used as the substrate, prepared according to the methodology described in the literature [29]. The enzymatic activity was determined using the DNS (3.5-dinitrosalicylic acid) method, which measures the concentration of reducing sugar in solution [30,31]. The reaction solution contained 200 μL of chitinase and 200 μL of 5% (*w*/*v*) colloidal chitin in a citrate-phosphate buffer (McIlvaine), pH 5.0 [32]. The mixture was incubated for 1 h at 50 °C under stirring at 250 rpm. Subsequently, 400 μL of 3,5-dinitrosalicylic acid was added, and the reaction mixture was heated for 10 min at 100 °C and then cooled to −20 °C for 5 min. The solution was centrifuged at 10,000× *g* for 5 min, and the supernatant was analyzed using a spectrophotometer (BIOMATE 160, Thermo Fisher Scientific, Waltham, MA, USA) at 540 nm. The tests were performed in triplicate, and the results were compared with a standard curve of N-acetylglucosamine (GlcNAc) ranging from 0.1 to 1 mg/mL.

Specific activity was defined as the amount of enzyme that released 1 μmol of GlcNAc per minute under optimal pH and temperature conditions.

### 2.5. Effects of pH and Temperature on Enzyme Activities

The optimum pH was determined by measuring chitinase activity at 50 °C in McIlvaine buffer ranging from pH 3.0 to 8.0. The reaction mixture consisted of 200 μL of chitinase and 200 μL of 5% (*w*/*v*) colloidal chitin prepared in McIlvaine buffer at the corresponding pH values. Samples were incubated and analyzed as previously described.

To determine the optimal temperature, enzymatic assays were conducted at the optimal pH for each chitinase over a temperature range of 25 °C to 80 °C.

To evaluate the thermal stability of the chitinases, the purified enzymes were pre-incubated at 4 °C, 28 °C, 37 °C, 50 °C, and 55 °C. The incubation periods were conducted at 1, 8, 16, 24, 48, and 72 h. Subsequently, the residual activity was measured under optimal conditions (pH and temperature) as previously described. The data were expressed as the mean absorbance of three replicates from three independent experiments, with the standard deviations indicated.

### 2.6. Sample Preparation for NMR Experiments

To identify and quantify the generated oligosaccharides from enzymatic reactions, aliquots from each reaction were analyzed by Nuclear Magnetic Resonance (NMR) spectroscopy. Aliquots of 350 μL of each sample was combined with 250 μL of deuterium oxide (D_2_O), containing an internal standard solution of sodium 3-trimethylsilylpropionate-d4 (0.50 mM, TMSP in D_2_O). Consequently, the final concentration of TMSP in the prepared solutions was 0.21 mM. Subsequently, the samples were carefully transferred to 5 mm NMR tubes and placed in the NMR spectrometer for analysis.

One-dimensional (1D) and two-dimensional (2D) NMR experiments were performed on a Bruker AVANCE III 14.1 T spectrometer (^1^H observed at 600.13 MHz, Bruker BioSpin, Rheinstetten, Germany), equipped with a 5 mm Broad Band Observe (BBO) probe featuring Automatic Tuning Matching Adjustment (ATMA) and a BCU-I variable temperature unit. All measurements were conducted at 298.15 K.

The ^1^H NMR spectra were acquired using a pre-saturation solvent suppression pulse sequence (noesypr1d) to suppress the water signal by irradiation applied at the frequency of 2823.57 Hz (O1). Acquisition parameters were as follows: 64 scans (ns), 4 dummy scans (ds), 65,536 data points (td), spectral width (sw) of 20.03 ppm, receiver gain (rg) of 64, 90° pulse (P1) of 12.11 μs, acquisition time (aq) of 2.73 s, and a 5 ms mixing time (d8).

All ^1^H spectra were processed using TopSpin 3.7 software (Bruker BioSpin GmbH, Rheinstetten, Germany). Chemical shifts were referenced to TMSP-d_4_ signal at 0.0 ppm. Prior to Fourier transformation, an exponential line broadening (lb) of 0.3 Hz was applied. Automatic phase correction and baseline adjustment were performed, and when necessary, the manual corrections were applied.

N-acetylglucosamine (GlcNAc) was identified in the ^1^H spectra by comparison with reference chemical shift data from the human metabolome; HMDB—Human Metabolome Database (N-acetylglucosamine) (HMDB0000215). The assignment was further confirmed using ^1^H-^1^H COSY NMR experiment. Additionally, GlcNAc signals in the ^1^H-NMR spectra was also identified using the Profiler module of the Chenomx NMR Suite Professional v10 software (Chenomx Inc., Edmonton, AB, Canada). Relative quantitation of GlcNAc was performed by comparing the area integral of selected GLcNAc peaks to the area integral of the TMSP methyl peak at 0.00 pm, which corresponded to a known concentration of 0.521 mM in all samples.

### 2.7. Antifungal Activity

To evaluate the antifungal activity of the three chitinases, two human pathogenic fungi were tested, *Aspergillus fumigatus* and *Candida albicans* (ATCC64548) as representatives of the WHO fungal priority pathogens list ranked in the critical priority group [33]. To monitor the growth of the wild-type *A. fumigatus*, 2.5 × 10^4^ conidia of the strain were cultivated in 200 μL of liquid YG medium (2% [wt/vol] glucose, 0.5% [wt/vol] yeast extract, 0.1% [*v*/*v*] trace elements), pH 6.5 [34], which were supplemented with varying concentrations of chitinases (100, 50 and 25 μg/mL). Each chitinase was evaluated individually, and additional assays were performed to investigate the potential synergistic effects among the chitinases. The dye resazurin was utilized at a final concentration of 0.002% (m/V) as the viability indicator [35]. The antifungal evaluation of the chitinase was performed after 24 to 30 h at 37 °C in black 96-well plates (Greiner Bio-One; 655.086, Frickenhausen, Germany). Following the incubation period, the percentage reduction in resazurin was determined by measuring the fluorescence at wavelengths of 570 nm (excitation) and 615 nm (emission) using a SpectraMax i3 plate reader (Molecular Devices, San Jose, CA, USA). The mean fluorescence values were used to calculate growth inhibition (%) of the fungus in the presence and absence of chitinases. To ensure consistency in the experimental conditions, the same procedure was conducted in the presence of clinical antifungal Amphotericin B (2 μg/mL). The percentage of resazurin reduction and growth inhibition was calculated using the methodology described [35]. Resazurin was used to monitor *Aspergillus* viability because it measures metabolic activity, which is suitable for filamentous fungi. Experiments were performed in quadruplicate, and data are expressed as the mean of the replicates.

For the *Candida albicans* assays, 2.5 × 10^4^ conidia were inoculated into 200 μL of liquid using Sabouraud dextrose liquid, supplemented with varying concentrations of chitinases (150, 100, and 50 μg/mL). Chitinase activity against *C. albicans* was assessed after 24 to 30 h of incubation at 37 °C. To evaluate the potential for synergistic activity among the chitinases, additional assays were performed in the presence of calcofluor white (CFW), a fluorescent agent that disrupts the fungal cell wall [36]. The growth of *C. albicans* cells was monitored by reading the optical density at OD_600_, a standard turbidity-based method commonly applied to unicellular yeasts. All the experiments were carried out in quadruplicate, and data expressed as the mean of the replicates.

The activity of the three enzymes against the plant pathogen *Lasiodiplodia theobromae* was evaluated in accordance with the methodology outlined in the literature [24]. In brief, an 8 mm mycelial sample of the fungus was cultivated on PDA medium and subsequently transferred to 10 mL of culture medium. The culture was incubated at 28 °C with 150 rpm agitation for 72 h. Subsequently, chitinases (0.71 mg) were added individually for each experiment. The culture was maintained for an additional 72 h under the same conditions to assess fungal growth. The experimental design included a positive growth control (without enzyme) and antifungal control (Amphotericin B (2 μg/mL)). All the experiments were carried out in triplicate from independent experiments.

For scanning electron microscopy (SEM) analysis, mycelial samples from the culture medium after 72 h of incubation with chitinase were fixed in Karnovsky solution (4% paraformaldehyde, 5% glutaraldehyde, 0.05% CaCl_2_) for 24 h at room temperature, followed by acetone dehydration. The samples were lyophilized, gold-coated and imaged using a JEOL JSM 6510 microscope (5 kV, WD 10 mm) (JEOL, Tokyo, Japan) to observe morphological changes in the fungal hyphae.

## 3. Results and Discussion

### 3.1. DNA Amplification and Analysis

**Figure 1:**
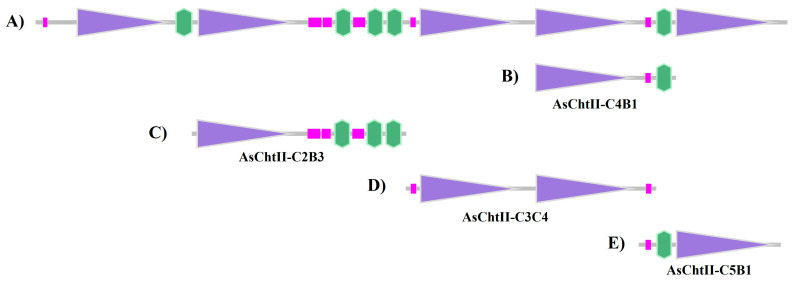
Analysis of the chitinases sequences based on the SMART program (http://smart.embl-heidelberg.de/, accessed on 15 January 2021). (**A**) Group II chitinase predicted sequence of *A. echinatior* (XM_011067337.1); (**B**) Construction corresponding to AsChtII-C4B1; (**C**) Construction corresponding to AsChtII-C2B3; (**D**) Construction corresponding to AsChtII-C3C4; (**E**) Construction corresponding to AsChtII-C5B1. Purple boxes: catalytic domains (Glyco_18); pink boxes: low complexity region; green boxes: chitin-binding module (CBM).

Since the genome of *Atta sexdens* has not yet been sequenced, primers were designed based on the deposited sequence of *Acromyrmex echinatior* (NCBI Reference Sequence: XM_011067337.1 (https://www.ncbi.nlm.nih.gov/nuccore/XM_011067337.1, accessed on 15 January 2021), another species of leaf-cutting ant. Translation of this sequence yields a polypeptide belonging to group II of the GH18 glycoside hydrolase family, composed of 2678 amino acid residues and containing five catalytic domains (C) and five chitin-binding modules (CBMs) (Figure 1A). In this figure, purple boxes represent catalytic domains (Glyco_18), pink boxes indicate low-complexity regions, and green boxes represent the chitin-binding modules (CBMs).

In a previous study, a polypeptide chain containing one catalytic site and one CBM in the C-terminal region (AsChtII-C4B1, Figure 1B) was obtained and characterized, which interfered with the growth of human and plant pathogenic fungi [23,24]. To evaluate the activity of different catalytic domains and chitin-binding modules (CBMs), three polypeptide constructs were designed: AsChtII-C2B3 (containing catalytic domain C2 and three CBMs at the C-terminus), AsChtII-C3C4 (containing catalytic domains C3 and C4), and AsChtII-C5B1 (containing catalytic domain C5 and one CBM at the N-terminus). (Figure 1C–E).

PCR products of the three constructs matched the expected sizes: AsChtII-C3C4 (3000 bp), AsChtII-C5B1 (1600 bp), and AsChtII-C2B3 (2400 bp) (Figure S1, Supplementary Materials). The DNA sequence was translated into protein sequences, with the characteristics briefly summarized in Table 1.

As expected, the three sequences share over 90% identity with chitinase sequences previously deposited for *Acromyrmex echinatior* and *Atta cephalotes.* The catalytic domain C4 contains the highly conserved motif FDGXDLDWEYP expected for a catalytic domain in the family 18 chitinases [13], while the C3 domain presents a methionine replacing the leucine in the motif (FDGXDMDWEYP) (Figure S2, Supplementary Materials), as observed in *A. cephalotes* and *A. echinatior* sequences. The C2 and C5 domains in *A. cephalotes*, *A. echinatior*, and in *A. sexdens* (this work) show some differences in this motif (Figures S3 and S4, Supplementary Materials). In the C5 domain, two substitutions result in the FDGLNLDWEFP motif in place of the FDGXDLDWEYP motif. The second D amino acid of the motif is also not conserved in the C2 domain, being replaced by a serine, and the glutamic amino acid, which has been reported to be fundamental for the activity of the enzyme, is replaced by a serine as well (FDGLSLDWSP).

Among the three chitinase constructs analyzed, AsChtII-C2B3 is predicted to lack catalytic activity due to the absence of the conserved glutamic acid residue in its C2 domain. This residue is also missing in the *Monochamus alternatus* chitinase sequence (GenBank: BAG13449.1), indicating potential evolutionary divergence. AsChtII-C3C4 (804 amino acids) contains two catalytic domains (C3 and C4) but lacks CBMs, while AsChtII-C5B1 (553 amino acids) exhibits a premature stop codon upstream of the His-tag, likely resulting from a PCR-induced mutation, which may affect protein expression and function (Figures S3 and S4, Supplementary Materials).

### 3.2. Obtaining the Recombinant Proteins

The polypeptide chains were heterologously expressed in *Pichia pastoris* using a secretion vector designed to produce the recombinant proteins in the extracellular medium. Ammonium sulfate precipitation (80% saturation) was employed as an initial step to concentrate the crude extract and reduce its volume. Following precipitation, the samples were dialyzed and applied to a Ni-NTA affinity chromatography column.

As expected, AsChtII-C5B1, which lacks the His-tag due to a premature stop codon, did not bind to the nickel resin and was recovered in the flow-through fraction. The His-tagged proteins, AsChtII-C2B3 and AsChtII-C3C4, were successfully eluted from the column using imidazole, dialyzed, and subsequently subjected to size-exclusion chromatography using a Superdex 75 column (Cytiva, Uppsala, Sweden). AsChtII-C5B1 was also purified using the same size-exclusion step.

Following purification, C2B3 and C3C4 showed high purity, whereas C5B1 exhibited slightly lower purity, likely due to the absence of a His-tag. The theoretical molecular masses, calculated based on the coding sequences including the His-tag using the ExPASy ProtParam tool (https://web.expasy.org/protparam/, accessed on 20 January 2021), were approximately 85 kDa for AsChtII-C2B3, 88 kDa for AsChtII-C3C4, and 60 kDa for AsChtII-C5B1 (Table 1). As observed in the SDS–PAGE analysis (Figure S5, Supplementary Materials), all recombinant proteins migrated with an apparent molecular weight higher than predicted, most likely due to glycosylation resulting from expression in *Pichia pastoris*.

### 3.3. Enzymes Biochemical Characterization

#### 3.3.1. Analysis of the GlcNAc Produced by ^1^H NMR

The enzymatic activity of the chitinases on colloidal α-chitin was monitored by ^1^H NMR Spectroscopy. This technique enables a rapid evaluation of chitinase activity, through the identification and quantification of resulting product, GlcNAc (Figure S6, Supplementary Materials). GlcNAc, was identified based on chemical shift, multiplicity and spin-spin coupling constants observed in the ^1^H NMR spectra (Table S2, Supplementary Materials). ^1^H-^1^H COSY NMR experiment (Figure S7, Supplementary Materials) was performed to confirm the assignment of GlcNAc. For the quantification of GlcNAc, signals corresponding to the hydrogen of the N-acetyl group (2.03 ppm) and the anomeric hydrogens of GlcNAc (5.19 ppm) were selected.

The spectra were recorded under similar conditions, and the concentrations of GlcNAc measured were 0.41, 0.48, and 0.56 mmol L^−1^ in the reactions catalyzed by AsChtII-C3C4, AsChtII-C2B3, and AsChtII-C5B1, respectively. These results demonstrate that the recombinant chitinases are active on colloidal α-chitin substrate. A previous study reported GlcNAc production of 2.60 mmol L^−1^ by AsChtII-C4B1 under similar reaction conditions [24]. This difference may reflect the distinct structural and catalytic features of each enzyme variant, consistent with studies showing that domain composition significantly influences chitinase activity and substrate affinity [16].

#### 3.3.2. Effect of pH on the Enzymatic Activity of AsChtII-C2B3, AsChtII-C3C4, and AsChtII-C5B1

**Figure 2:**
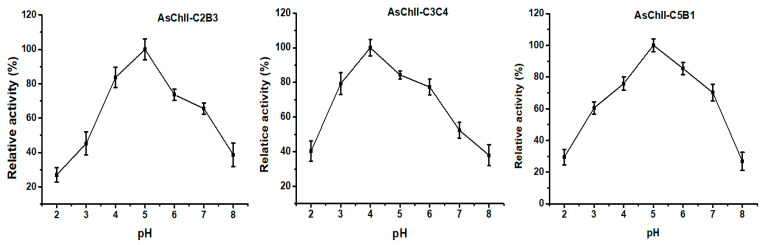
Effects of pH on the activities of chitinases AsChtII-C2B3, AsChtII-C3C4 and AsChtII-C5B1. The activity of each enzyme was measured at 50 °C on colloidal α-chitin as substrate. The highest activity of each enzyme was defined as 100%. The graphs show data from triplicate experiments (mean ± SD).

The effect of pH on chitinase activity was investigated by testing the enzymes over a broad pH range (3.0 to 8.0) using the DNS (3.5-dinitrosalicylic acid) method. The three recombinant chitinases studied, AsChtII-C2B3, AsChtII-C3C4, and AsChtII-C5B1 demonstrated optimal activity within the acidic pH range of 4.0 to 5.0. Specifically, AsChtII-C2B3 and AsChtII-C5B1 exhibited peak activity at pH 5.0, while AsChtII-C3C4 showed its highest activity at pH 4.0 (Figure 2). All three chitinases maintained approximately 80% of their maximum activity within a ±1 pH unit range around their respective optima, indicating moderate pH stability.

These findings are in agreement with previously reported pH optima for insect chitinases, which typically display activity between pH 4.0 and 8.0 [37]. Group I chitinases, such as those from *Ostrinia furnacalis*, have shown optimal activity at pH 6.0–6.5 [38,39], whereas the recombinant rEF-Chi enzyme maintained high activity between pH 5.0 and 8.0 [40]. Chitinases of varying sizes from *Manduca sexta* also exhibited activity within this range [41]. Importantly, the group II chitinases OfChtII-C1 and OfChtII-C2 from *O. furnacalis* displayed optimal activity at pH 6.0 [16] while the previously studied AsChtII-C4B1 from *Atta sexdens* showed strong activity at pH 5.0 [23].

#### 3.3.3. Effect of Temperature and Thermostability of the Chitinases

**Figure 3:**
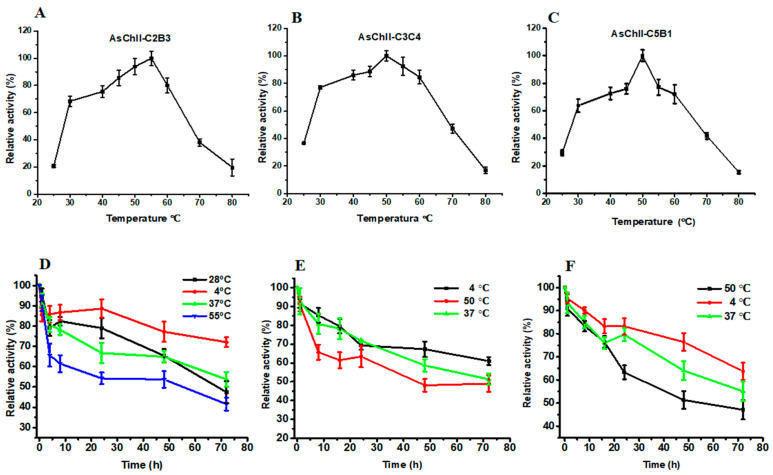
Effects of temperature on enzymatic activity and thermostability of chitinases AsChtII-C2B3, AsChtII-C3C4, and AsChtII-C5B1. (**A**–**C**) Effect of temperature on the relative activity of AsChtII-C2B3 (**A**), AsChtII-C3C4 (**B**), and AsChtII-C5B1 (**C**), measured at temperatures ranging from 25 to 80 °C. (**D**–**F**) Thermostability of AsChtII-C2B3 (**D**), AsChtII-C3C4 (**E**), and AsChtII-C5B1 (**F**), determined by measuring residual activity after incubation at different temperatures (4, 28, 50, 37, and 55 °C) for up to 72 h. Relative activity is expressed as a percentage of the initial activity (100%). The activities of each enzyme were measured at its optimal pH with colloidal α chitin as the substrate. The highest activity of each enzyme was defined as 100%. The graphs show data from triplicate experiments (mean ± SD).

The enzymatic activities of the three recombinant chitinases increased progressively with temperature, reaching their peak between 50 °C and 55 °C. AsChtII-C2B3 showed optimal activity at 55 °C (Figure 3A), while AsChtII-C3C4 (Figure 3B) and AsChtII-C5B1 (Figure 3C) had maximal activity at 50 °C.

Thermostability was assessed by incubating the enzymes at various temperatures. AsChtII-C2B3 retained approximately 53% of its activity after 48 h at 55 °C and around 65% at 28 °C and 37 °C. For AsChtII-C3C4 and AsChtII-C5B1, enzymatic activity declined to approximately 48% and 51%, respectively, after 48 h at 50 °C. Both also maintained similar stability at 37 °C.

All three enzymes retained considerable residual activity even after 72 h at their optimal temperatures. Additionally, they exhibited the greatest stability at 4 °C throughout the entire incubation period.

These findings suggest that the recombinant chitinases possess moderate thermostability, which may be advantageous for applications involving prolonged exposure to moderately elevated temperatures. Compared to the previously studied AsChtII-C4B1, which also exhibited optimal activity at 55 °C but showed reduced stability after 48 h at this temperature [23]. The partial constructs characterized here showed similar thermal profiles, reinforcing the stability potential of AsChtII chitinases for biotechnological use.

The specific activity of an enzyme is a critical parameter in understanding its catalytic efficiency under optimal conditions. A common method to assess chitinase activity is the dinitrosalicylic acid (DNS) assay, which quantifies reducing sugars [30].

The recombinant enzymes were evaluated against colloidal chitin under optimal conditions using the DNS method. The amounts of enzyme required to release 1 μmol of GlcNAc per minute were 22, 52, 68, and 88 nmol for AsChtII-C4B1, AsChtII-C2B3, AsChtII-C5B1, and AsChtII-C3C4, respectively. These values reflect the enzyme quantity needed under the assay conditions. However, lower amounts indicate that an enzyme is more effective under these specific conditions, allowing relative comparison of their efficiency in the assay (Table 2). This activity trend is consistent with that observed in the NMR analysis (Section 3.3.1). AsChtII-C4B1, which retains the conserved FDGXDLDWEYP motif in its catalytic domain, displayed the highest activity, reinforcing the functional importance of this motif.

Notably, AsChtII-C3C4, which comprises two catalytic domains (C3 and C4) but lacks a chitin-binding module (CBM), exhibited the lowest activity in both DNS and NMR assays. The C3 and C4 domains share 70% sequence identity, suggesting that structural redundancy without proper CBM support or synergistic conformation may not enhance catalytic efficiency. Meanwhile, AsChtII-C2B3, despite containing three CBMs, showed lower activity than AsChtII-C4B1, likely due to key mutations in its catalytic motif. These findings suggest that while CBMs can enhance substrate interaction, the presence of conserved catalytic residues is essential for efficient catalysis. However, in the present study, neither the presence of multiple catalytic domains nor the number of CBMs consistently correlated with increased enzymatic activity.

The enzymes exhibited consistent activity across the conditions evaluated, suggesting that they are unlikely to be readily inactivated under environmentally relevant conditions, including those found in plant tissues or soil. This stability supports their potential effectiveness, although further in situ studies will be necessary to validate their performance in complex biological environments.

### 3.4. Analysis of the Chitinase Activity on Fungal Growth and Susceptibility

#### 3.4.1. Activity Against Human Pathogenic Fungi

The three recombinant chitinases were evaluated for their antifungal activity against two clinically relevant pathogenic fungi, *Candida albicans* and *Aspergillus fumigatus* to cover the spectrum of action over yeast and filamentous fungal pathogens.

*C. albicans* exists as a commensal on human mucosal surfaces, and it has the potential to transition to a pathogenic state, following adhesion and fungal overgrowth, which can then result in tissue invasion and mucosal infection [42].

The antifungal efficacy of the recombinant chitinase constructs was assessed against *C. albicans* by measuring fungal growth inhibition at increasing enzyme concentrations (50, 100, and 150 µg/mL), both with and without the addition of Calcofluor White (CFW). CFW is a fluorescent dye that binds to chitin in the fungal cell wall, disrupting its assembly [36], and may potentially enhance the effects of chitinolytic enzymes via synergistic interaction.

**Figure 4:**
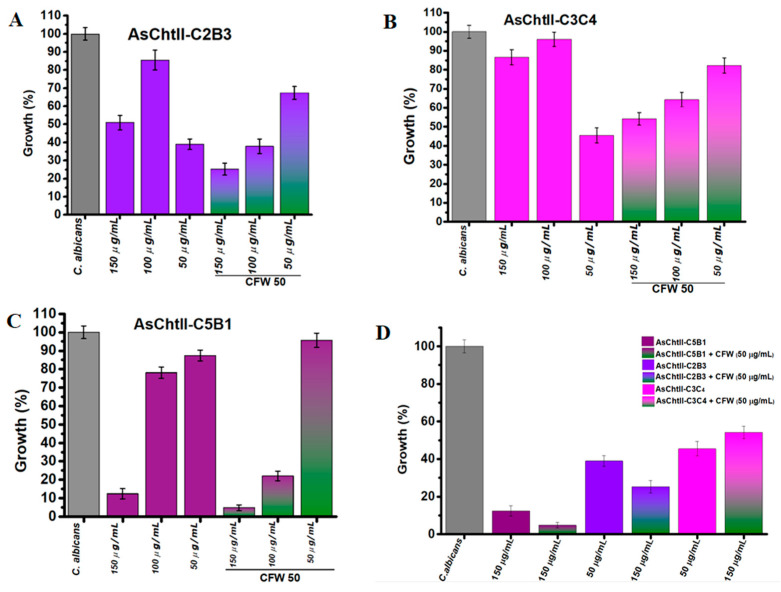
Interference of chitinases in the growth of *C. albicans* in the presence and absence of CFW. (**A**–**C**) *C. albicans* growth (%) after treatment with individual chitinase variants at different concentrations (50, 100, 150 µg/mL) alone or in combination with 50 µg/mL CFW. (**D**) Summary of all chitinase variants: Chitinase alone (AsChtII-C5B1, AsChtII-C2B3, AsChtII-C3C4) and in combination with CFW (50 µg/mL). The control *P. pastoris* KM71H transformed with an empty pPICZαA was tested under the same conditions, and the values obtained for each dilution were subtracted from the values obtained for the chitinases. The results presented were obtained from the mean of the quadruplicate for each sample, with standard deviations included.

AsChtII-C2B3 exhibited moderate antifungal activity that was not dose-dependent. In the absence of CFW, fungal growth was reduced by 47% at 150 µg/mL and 61% at 50 µg/mL, indicating a non-linear response. When CFW was added, the inhibitory effect was enhanced, with fungal growth reduced to 60% at 100 µg/mL, and 75% at 150 µg/mL (Figure 4A).

AsChtII-C3C4 showed weakest antifungal activity when compared to AsChtII-C2B3. Without CFW, inhibition was modest, 54.5% at the lowest concentration (50 µg/mL), again indicating non-dose-dependent behavior. The addition of CFW slightly improved inhibition, with reducing fungal growth to 55% (150 µg/mL) and 45% (100 µg/mL) (Figure 4B). The weaker inhibition of *C. albicans* growth observed at 50 µg/mL with CFW, when compared to higher enzyme concentrations, suggests that at lower enzyme levels, CFW may bind to the chitin substrate in a manner that obstructs the catalytic access sites of AsChtII-C3C4, hereby limiting effective enzyme hydrolysis.

In contrast, AsChtII-C5B1 demonstrated the strongest antifungal effect against *C. albicans*. In the absence of CFW, the percentage of fungal growth was reduced by 88% at 150 µg/mL, indicating potent activity. The presence of CFW further enhanced this effect, reducing the growth of *C. candida* to 95% at 150 µg/mL (Figure 4C). These results suggest a clear synergistic interaction between CFW and C5B1 that can be translated into potent chitinase activity facilitated by the disorganization of the fungal cell wall caused by the cell wall-stressing agent CFW, a feature that can be shared with other cell wall stressors.

When the constructs were compared under their best performance conditions (Figure 4D) (150 µg/mL + CFW), AsChtII-C5B1 consistently outperformed AsChtII-C2B3 and AsChtII-C3C4. This reinforces the superior efficacy of C5B1 and demonstrates that both enzyme structure and enzyme–cell wall stressor interaction influence antifungal performance.

In a previous study, *C. albicans* displayed no detectable growth inhibition when exposed to AsChtII-C4B1 alone at any tested concentration, and only a subtle synergistic effect was observed in combination with higher doses of CFW (200 µg/mL), with no enhancement at lower CFW concentrations [23].

Because the catalytic domain (CAT) and chitin-binding modules (CBMs) are critical structural components of chitinases, we compared the deduced amino acid sequences of these domains to investigate possible correlations with antifungal activity. The chitinases showed low sequence identity between their catalytic domains. For example, the C2 domain of AsChtII-C2B3 shares only 36% identity with the C5 domain of AsChtII-C5B1, 43% with C3, and 45% with C4 of AsChtII-C3C4. The C3 and C4 domains of AsChtII-C3C4 exhibit higher similarity (69%), reflecting partial conservation within this tandem construct.

Interestingly, despite the relatively low sequence identity (45%) between the catalytic domains of AsChtII-C2B3 and AsChtII-C3C4, both constructs displayed comparable antifungal activity, inhibiting *C. albicans* by 61% and 54.5%, respectively, at 50 µg/mL. This suggests that functional conservation can occur despite limited sequence similarity, possibly due to conservation of key active-site residues or tertiary structural elements critical for chitin hydrolysis.

The presence of two catalytic domains in AsChtII-C3C4 did not lead to enhanced antifungal activity. On the contrary, its performance was lower than that of the single-CAT construct AsChtII-C2B3. One possible explanation is that the two CATs in C3C4, linked by a flexible region, may adopt nonproductive binding positions that hinder effective substrate engagement. Moreover, C3C4 lacks chitin-binding modules, which may further reduce its affinity for fungal cell wall chitin and limit overall activity.

In contrast, AsChtII-C2B3 contains a single CAT domain and three CBMs at its C-terminal region. While this construct inhibited fungal growth by 61%, it remains unclear whether all three CBMs contribute equally to the observed activity. CBMs are typically known to enhance catalytic efficiency by promoting binding to the polysaccharide substrate [43], but the functional roles and spatial arrangement of multiple CBMs in this construct warrant further investigation.

The role of CBMs is more clearly illustrated in AsChtII-C5B1, which contains a single CAT and one CBM, yet displayed the highest antifungal activity (88% inhibition at 150 µg/mL + CFW). This result suggests that a balanced combination of an efficient catalytic core and a strategically placed CBM may be more effective than multiple domains without optimal spatial orientation or substrate accessibility.

Overall, these findings emphasize that chitinase antifungal activity in this case is influenced not only by the number of catalytic or binding domains, but also by their structural organization, domain synergy, and accessibility to the fungal cell wall substrate.

We extended our analysis to wild-type *A. fumigatus*, a saprophytic fungus, commonly found in soil, regions with a subtropical or warm temperate climate [44]. The fungus reproduces asexually by producing hydrophobic conidia, which can reach the alveoli in the lungs. It is estimated that 2,113,000 people suffer from invasive aspergillosis in the context of chronic obstructive pulmonary disease, intensive care, lung cancer, or hematological malignancy, with a crude annual mortality of 1,801,000 (85.2%) [45]. The range of human infections caused by the fungus is broad, from allergic diseases to invasive aspergillosis, depending on the immune status of the host [46].

The antifungal activity of individual AsChtII chitinases was evaluated at three concentrations (100, 50, and 25 µg/mL) to determine their effectiveness in inhibiting *A. fumigatus* growth. Amphotericin B (2 µg/mL) was used as a positive control for fungicidal activity.

**Figure 5:**
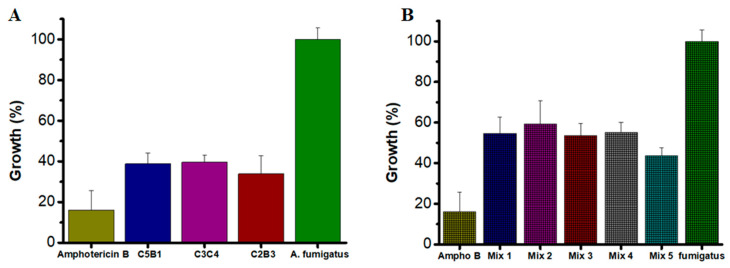
Growth of *A. fumigatus* in the presence of recombinant chitinases. (**A**) Individually: AsChtII-C5B1 and AsChtII-C2B3 at 50 µg/mL, AsChtII-C3C4 at 25 µg/mL (**B**) Synergistic interactions between chitinase. The control *P. pastoris* KM71H transformed with an empty pPICZαA was tested under the same conditions, and the values obtained for each dilution were subtracted from the values obtained for the chitinases. The results presented were obtained from the mean of the quadruplicate for each sample, with standard deviations included.

All tested enzymes demonstrated significant inhibitory effects, with growth reductions exceeding 60% at their optimal concentrations (Figure 5A). Specifically, at 50 µg/mL, AsChtII-C2B3 and AsChtII-C5B1 inhibited fungal growth by 66% and 61%, respectively. AsChtII-C4B1, previously characterized at 50 µg/mL, exhibited an inhibition rate of approximately 67% [23]. Notably, AsChtII-C3C4 achieved its highest inhibitory activity of 60% at the lower concentration of 25 µg/mL, suggesting in this organism this enzyme can more effectively interact with the solid chitin matrix of the fungal cell wall even in the absence of a carbohydrate-binding module (CBM). For comparison, Amphotericin B, a well-established antifungal agent, reduced fungal growth by approximately 84%, confirming the robustness of the assay and the relative potency of the chitinases tested.

To explore potential synergistic interactions, combinations of chitinases were tested at the lowest concentration previously evaluated (25 µg/mL). The combinations varied in their inhibitory effects, with Mix 5 (AsChtII-C2B3 and AsChtII-C5B1) achieving inhibition of 56.2% (Figure 5B). Other enzyme mixes showed moderate inhibition, ranging from approximately 40% to 46%. While these results suggest some degree of synergy, the overall enhancement was limited compared to the activity of individual enzymes at higher concentrations.

The constrained synergistic effect observed may be attributed to several factors. First, *A. fumigatus* likely mounts a compensatory response to enzymatic degradation by overproducing chitin, thereby reinforcing its cell wall and diminishing the combined efficacy of chitinase mixtures. This adaptive remodeling aligns with the paradoxical effect reported in fungal responses to cell wall-targeting antifungals such as the echinocandin caspofungin [47,48,49]. The caspofungin paradoxical effect has been widely associated with increased chitin content in the cell wall due to a compensatory upregulation of chitin synthase-encoding genes [47]. Second, the fungal cell wall’s complex architecture and heterogeneous composition may restrict substrate accessibility, limiting cooperative enzyme activity. Additionally, competition among chitinases for substrate binding sites and overlapping catalytic specificities can prevent effective synergy, especially at higher enzyme concentrations where substrate saturation occurs.

Collectively, these findings underscore the complexity of chitinase-mediated antifungal activity and highlight the importance of enzyme selection, concentration, and fungal adaptive mechanisms in optimizing synergistic interactions. Future studies should investigate the molecular basis of the fungal adaptive response and systematically characterize enzyme kinetics in combination to better harness the potential of chitinase synergy.

#### 3.4.2. Activity Against Phytopathogen Fungi

*L. theobromae* is a pathogen with a wide geographic distribution that is responsible for causing over 500 plant diseases, including fruit root, root rot, collar rot, dieback, canker, and leaf necrosis [50,51]. In humans, infection by this phytopathogenic fungus is relatively uncommon; however, it has been associated with a range of clinical manifestations, including fungal infection of the nails, corneal ulcers, rhinosinusitis, and mycosis in immunodeficient patients [52,53]. Therefore, chitinases were evaluated for their ability to interfere with the growth of this phytopathogen fungus. Among them, only AsChtII-C2B3 exhibited a positive antifungal effect. Interestingly, the C2 catalytic site of this enzyme shows a poorly conserved motif, with mutations in amino acids previously reported as essential for activity. Despite this, AsChtII-C2B3 was able to inhibit fungal development, suggesting that in this case the presence of a CBM may play a critical role in mediating antifungal activity.

**Figure 6:**
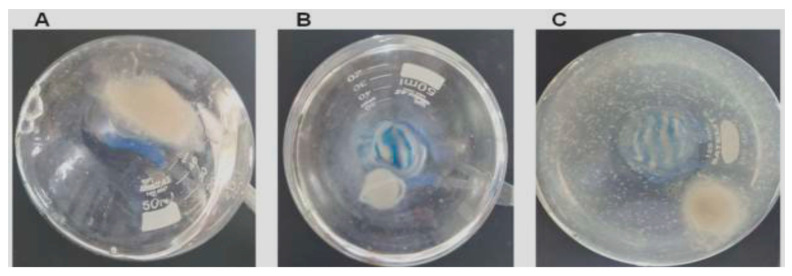
AsChII-C2B3 activity on the growth of fungus *L. theobromae* in liquid media. (**A**) fungal growth in the absence of the enzyme, (**B**) fungal growth in the presence of commercial fungicide Amphotericin B, and (**C**) fungal growth in the presence of the AsChII-C2B3. Each experiment was conducted with a minimum of three independent replicates.

After treatment, suspended solids mycelial clumps, likely representing hyphal fragments, were observed in the culture medium (Figure 6C), and the overall fungal mycelial mass appeared considerably diminished compared to untreated controls (Figure 6A).

To evaluate fungal viability, samples of the culture medium with digested hyphae were inoculated on BDA medium and incubated at 28 °C for 72 h, and no fungal colonies were found from cultures treated with AsChtII-C2B3, suggesting a loss of viability under these circumstances.

**Figure 7:**
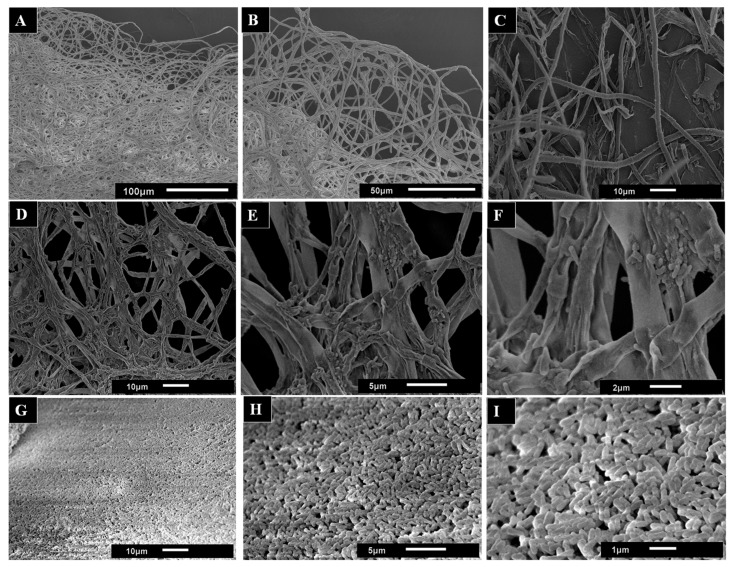
SEM images used to observe the morphological changes in the hyphae of the fungus *L. theobromae* scored under the activity of the recombinant chitinase AsChtII-C2B3. (**A**,**B**) hyphae of the fungus in the absence of the enzyme. (**C**–**I**) hyphae of the fungus treated with the enzyme. Morphological alterations of the hyphae, such as crushing of the tubular parts, long holes on the surface, and swollen tubes, can be observed. The images in (**G**–**I**) illustrate samples of the hyphae extracted from the medium. The images demonstrate the presence of minute tubes that bear resemblance to digested hyphae.

Mycelium samples from liquid cultures were analyzed by scanning electron microscopy (SEM) in order to better understand the effect of AsChtII-C2B3 on the fungal cell wall. SEM images of untreated mycelia displayed a dense network of long tubular hyphae with smooth, uniform surfaces (Figure 7A,B). In contrast, the SEM images mycelia treated with AsChtII-C2B3 appeared brittle and thinner, with hyphae showing wrinkling, collapse, and roughened surfaces (Figure 7C–F). In addition, discontinuity of the tubular walls and extensively digested hyphae were evident in SEM images (Figure 7G–I).

The morphological alterations in *L. theobromae* hyphae caused by AsChtII-C2B3 are reminiscent of changes previously reported for AsChtII-C4B1, which also inhibited fungal growth. However, differences were observed between the enzymes; while AsChtII-C4B1 primarily disrupted hyphal cell walls by degrading nascent and mature chitin [23], exposure to AsChtII-C2B3 resulted in more extensive disorganization and hyphal collapse, suggesting possible preferential degradation of mature chitin. To confirm this specificity, further biochemical studies will be necessary.

The structural mechanism responsible for hyphal deformation and fungal inhibition by chitinases involves the hydrolysis of chitin in the fungal cell wall. Chitin, a major structural component, is often associated with β-glucans (1/3 and 1/6 linkages) and other polysaccharides, whose composition and cross-linking vary among species [10,54]. Most pathogenic fungi contain 5–27% chitin in their cell walls, with higher concentrations at hyphal tips and during mitosis [55], with species-specific differences (*Aspergillus fumigatus* ~20%, *Candida albicans* 5–7%, yeast 1–2%) [54,56]. Hydrolysis of chitin compromises cell wall integrity, causing abnormal hyphal morphologies such as swelling, branching, or distortion, and ultimately inhibiting fungal growth. The antifungal sensitivity of chitinases is influenced not only by the proportion of chitin but also by its accessibility, spatial distribution at hyphal tips or septa, and its association with other wall polymers, explaining the observed species-specific antifungal activity [10,54].

Although the precise structural determinants of the species-specific antifungal activity of the ant-derived chitinases remain uncharacterized, differences in domain architecture, enzyme stability, and substrate accessibility are likely contributors. Together with variations in fungal cell wall composition, including chitin content and its association with β-glucans, these factors provide a plausible explanation for the differential antifungal effects observed among fungal species, although further structural and kinetic analyses will be required.

Comparatively, chitinases from silkworm (*Bombyx mori*) and bollworm (*Helicoverpa armigera*) have been reported to inhibit fungal growth of *Saccharomyces cerevisiae* and *Penicillium* species by blocking germ tube elongation and causing hyphal deformation [57], while microbial chitinases from *Streptomyces sampsonii* and *Bacillus* spp. similarly reduce fungal growth and disrupt mycelial structure on four forest pathogens [58]. Plant-derived chitinases, such as from cashew (*Anacardium occidentale*), also induce hyphal disruption and inhibit fungal proliferation [59]. Taken together, these comparisons indicate that ant-derived chitinases exhibit comparable antifungal potency and mechanisms to those reported for other insect, microbial, and plant chitinases, effectively inducing hyphal deformation and growth inhibition across multiple fungal species, with some species-specific variability.

Apart from their direct antifungal effects, chitinases can act synergistically with fungicides and pesticides, enhancing their efficacy while reducing the amount of chemicals needed. This helps to minimize possible effects on the environment and health [60]. These results highlight that the assessment of chitinases as antifungal agents must take into account variations in fungal cell wall structure and point out potentialities to improve efficacy across wide a range of fungal pathogens via rational enzyme combinations or co-application with conventional antifungals.

## 4. Conclusions

This study reports the successful cloning, heterologous expression, and biochemical characterization of three insect-derived chitinases from the leaf-cutter ant *A. sexdens*. The recombinant enzymes displayed distinct structural domain arrangements and species-specific antifungal activities, demonstrating functional diversity among chitinases of insect origin. All enzymes exhibited optimal catalytic activity under acidic conditions and moderate temperatures, consistent with potential application in biotechnological processes. All three recombinant chitinases were able to interfere with the growth of the fungi tested. AsChtII-C5B1 showed pronounced antifungal activity against *C. albicans* while AsChII-C3C4 inhibited the growth of the *A. fumigatus* by approximately the same percentage but a requiring half its concentration. AsChtII-C2B3 was the only one to affect the growth of the phytopathogen *L. theobromae*, inducing severe hyphal deformities. These findings provide new insights into the molecular and functional diversity of insect chitinases and suggest that *A. sexdens* enzymes represent promising biocatalysts for use in medical and agricultural biotechnology. Future studies should explore their structural determinants of specificity and assess their performance in applied systems, such as biocontrol formulations and antifungal bioproducts.

## Figures and Tables

**Table 1:** Relevant characteristics of the three proteins AsChtII-C2B3, AsChtII-C3C4, and AsChtII-C5B1.

Protein	Amino Acids	Calculated Molecular Weight (kDa)	Catalytic Domains	CBM	Identity with *A. echinatior* (%)	Identity with *A. cephalotes* (%)
AsChtII-C2B3	771	85	C2	yes	93%	94%
AsChtII-C3C4	804	88	C3, C4	No	98.5% (C3), 98.8% (C4)	99.3% (C3), 98.5% (C4)
AsChtII-C5B1	553	60	C5	yes	95.7%	90.1%

**Table 2:** Properties of the chitinases from *A. sexdens* obtained in this study and in the previous literature.

Protein	Optimal pH	Optimal Temperature (°C)	Specific Activity * (nmol Enzyme)
AsChtII-C4B1 **	5.0	55	22
AsChtII-C2B3	5.0	55	52
AsChtII-C5B1	5.0	50	68
AsChtII-C3C4	4.0	50	88

* Quantity of enzyme that released 1 μmol of GlcNAc per minute under the optimal pH and temperature. ** Described in reference [23].

## Data Availability

Data are contained within the article and Supplementary Materials. Further inquiries can be directed to the corresponding author.

## Supplementary Materials

The following supporting information can be downloaded at https://www.mdpi.com/article/10.3390/polym18030402/s1, Figure S1: Analysis of the PCR products by agarose gel; Figure S2: AsChtII-C3C4 deduced amino acid sequence; Figure S3: AsChtII-C2B3 deduced amino acid sequence; Figure S4: AsChtII-C5B1 deduced amino acid sequence; Figure S5: SDS-PAGE of the proteins expressed *P. pastoris*; Figure S6: GlcNAc structure chemical; Figure S7: COSY ^1^H-^1^H NMR spectrum for GlcNAc; Table S1: Oligonucleotides to obtain the ORFs; Table S2: Assignments of the ^1^H NMR spectra in D_2_O for the GlcNAc.
