# Supplementary material for: Functional Characterization and Antifungal Activity of Insect-Derived Chitinases Expressed in Pichia pastoris
Source: Polymers (Basel). 2026 Feb 3;18(3):402. doi: 10.3390/polym18030402 (PMC12899241; doi:10.3390/polym18030402)
Supplement: Supplementary file 1 [file polymers-18-00402-s001.zip › polymers-4010496-supplementary.pdf]

Functional Characterization and Antifungal Activity of Insect-Derived Chitinases Expressed in *Pichia pastoris*

Katia Celina Santos Correa [a], Gabriel Henrique Ribeiro [b], Odair C. Bueno[c], Luiz Alberto Colnago [b], Iran Malavazi [d], Dulce Helena Ferreira Souza [a]

[a] Department of Chemistry, Federal University of Sao Carlos, São Carlos, SP, Brasil.

[b] Brazilian Agricultural Research Corporation (Embrapa Instrumentation), São Carlos, SP, Brasil.

[c] Center for the Study of Social Insects, Sao Paulo State University ´Julio de Mesquita Filho`, Rio Claro, SP, Brazil.

[d] Department of Genetics and Evolution, Federal University of São Carlos, São Carlos, SP, Brazil.

*Corresponding author:
Dulce Helena F. Souza
E-mail address: dulce@ufscar.br
Phone number: +55 16 3351 8074
Department of Chemistry, Federal University of São Carlos
Rd. Washington Luis, km 235, CEP – 13565-905
São Carlos - SP, Brasil

Caption	Page
Table S1 - Oligonucleotides to obtain the ORFs.	S3
Table S2 - Assignments of the ^{1}H NMR spectra in D_2O for the GlcNAc	S3

Captions	Page
**Figure S1** - Analysis of the PCR products by agarose gel electrophoresis	S4
**Figure S2** - AsChtII-C3C4 deduced amino acid sequence.	S5
**Figure S3** - AsChtII-C2B3 deduced amino acid sequence.	S6
**Figure S4** - AsChtII-C5B1 deduced amino acid sequence.	S7
**Figure S5** –SDS-PAGE of the proteins AsChtII-C2B3 (A), AsChtII-C3C4 (B), and AsChtII-C5B1 (C), expressed in *P. pastoris*.	S8
**Figure S6** - GlcNAc structure chemical	S9
**Figure S7** - COZY 1H-1H NMR spectrum for GlcNAc.	S10

Table S1 - Oligonucleotides to obtain the ORFs.
In red nucleotides of the restriction sites

Primers	Sequene 5' → 3'	Restriction enzyme
C2B3_F_S	**GATCCRADAGAGRAACGCTG**	
C2B3_R_S	**GACGATACCAAGCCCARTTGG**	
C2B3_F	**GCCGAATTCCATCATCATCATCATCATCCGCGTATAGCGTGTTATATGACG**	***EcoR* I**
C2B3_R	**GCCGCGGCCGCTCAGTCTGCGCATTTCGCCCGGG**	***Not* I**
C2B3_F_I	**CATGACGTACGACTATCATGGATC**	
C2B3_R_I	**GTAACTTTGGCGGTTGTAGTAGATG**	
C3C4_F_S	**GGAGAGTGTTTACGCTAAGGGC**	
C3C4_R_S	**GAGATCTTACCGAAGTTGCACAGATAATAC**	
C3C4_F	**GGCCCAGCCGGCCAGATGGCGACAAGGAAATTAC**	***Sfi* I**
C3C4_R	**GCCGGTACCATTGGAGGTAGTGGGCTGGCTTCGATC**	***Kpn* I**
C3C4_F-I	**GAATTGTAACAAGGGACCAGATTC**	
C3C4_R_I	**CGTTTAATCCGTGTACGTTTCTCTC**	
C5B1_F_S	**GCAGAATTAATCAAGAAACTTGGCCTC**	
C5B1_R _S	**AATGCAGTCTGTTGTGTGACCGTTCCC**	
C5B1_F	**GGGAATTCGACTTTAAGAACAGATGTGG**	***EcoR* I**
C5B1_R	**AAGCGGCCGCAATGCAGTCTGTTGTGTG**	***Not* I**
C5b1_R_I	**GGTAAGATCTCGGAACAGTCCTG**	

Tabe S2 – Assignments of the ^{1}HNMR spectra in D_2O for N-acetyl-D-dglucosamine compound. Chemical shifts (ppm), multicity, and coupling constants (Hz)

No.	^{1}H
1'	3.88 (m)
2'	5.19 d (2.8 Hz)
3'	3.40 – 3.95 (m)
4'	3.40 – 3.59 (m)
5'	3.40 – 3.95 (m)
6'	3.40 – 3.95 (m)
NH	8.09 d (8.0 Hz)
7'	-
8'	2.03 s

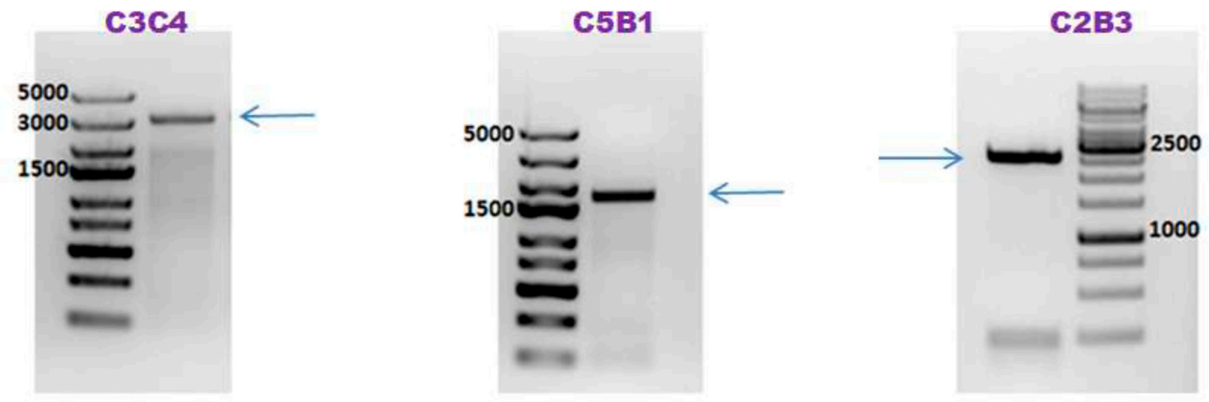

Figure S1 – Analysis of the PCR products by agarose gel. DNA fragments with the expected sizes of the about 3,000 bp for AsChtII-C3C4, 1,600 bp for AsChtII-C5B1 and 2, and 2,400 bp for AsChtII-C2B3.

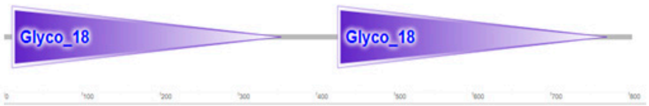


```
        10         20         30         40         50         60
QPARWRQGNY KVVCYFTNWA WYRRGIGRYL PEHIDHTLCT HIVYGFAVLD YSEMIIKAHD

        70         80         90        100        110        120
SWADFDNRFY ERVVAYKKRG LKVLLALGGW NDSAGDKYSR LVNNPSSRKK FINHTLQFIK

       130        140        150        160        170        180
KYDFDGLDMD WEYPVCWQVN CNKGPDSDKE SFAALLRELS AEFKPKGLLL SAAVSPSKKV

       190        200        210        220        230        240
IDKGYDVPSL AKYLDWIAVM AYDYHGQWDK RTGHVAPLYY HPDDEFYYFN ANYSINYWIS

       250        260        270        280        290        300
KGAPPRSIVM GMPLYGQSFT INDPSSGNGL NSPASAGNAG EFTRAAGFLS YYEICDRILN

       310        320        330        340        350        360
RGWTVVQDPE GRMGPYAYKG SQWVSFDDSE MIRRKAQFVR DMGLGGMVW ALDLDDFRGR

       370        380        390        400        410        420
CDNGPHPLMH TIQRVLAEPP KEHDKPLQPP SAPSPSITTL IVEPTQAIRP TNTVDRVTQN

       430        440        450        460        470        480
SFKNDEFKVI CYFTNWAWYR QEGGRFVPED IDPDLCTHVL YGFSVLDGSS LTMKSHDPWA

       490        500        510        520        530        540
DIDNKFYDRV AAFKAKGLKV LMALGGWNDS AGDKYSRLVN SPSARRKFIT QLLLFIEKYD

       550        560        570        580        590        600
FEGLDLDWEY PVCWQVDCNK GPESDKQSFA ELIKELSDEF KPRGLLLSAA VSPSKRVIDA

       610        620        630        640        650        660
GYDVPTLSKY LDWISIMTYD FHGQWDKKTG HVAPLYSLPN DWEPTFNANF SIHYWIEKGA

       670        680        690        700        710        720
NPKKLIMGVP LYGQSFSLAE RNVHGLNAPT YGGGEAGEAT RARGFLSYYE ICERTLKKGW

       730        740        750        760        770        780
TVVQDKERRI GPYAYKGDQW VSFDDAQQIK LKAELIKKLD LGGGMIWALD LDGTSSRGGR

       790        800
QPFWNKNSSQ KRIWNSAVDH HHHH
```

Figure S2 - AsChtII-C3C4 deduced amino acid sequence. Conserved motifs in the glycoside hydrolase 18 family chitinases are underlined in red. The predicted sites for N-glycosylation are highlighted in dotted boxes. The analysis of the sequence architecture of the sequence was developed using the SMART program (http://smart.embl-heidelberg.de/).

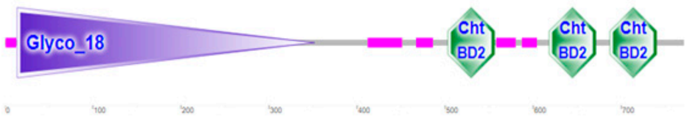


```
        10         20         30         40         50         60
EAEAAEFHHH HHHPRIACYM TSWALYRKGD GKFVPEHLDS NLCTDIIYAF AGLNPETLLI

        70         80         90        100        110        120
QPFDPWADIE NNLYQRVTSI KGSRILLALG GWTDSSGDKY SRLISSGVSR RKFVIATMNF

       130        140        150        160        170        180
LKKHNFDGLS FEWSYPKCWQ SDCKKGPDSD KPNFTKLMQE LREEFNKQDP PLLLAAALSG

       190        200        210        220        230        240
YKEVIDKAYE VHEISQIVDF MSVMTYDYHG SWESKLAHIA PLFSSSDSKP YYNVNFTMNY

       250        260        270        280        290        300
LIGGLGAEKS KLLVGIPLYG QSNRLSTASQ ADLGDPTTGP GKPGSSLSPG GWGRIMIFEK

       310        320        330        340        350        360
RLRHNWKVGI GPSAHFEDEW VGYEDRESVY ARGKYITEYG YGGATMWTID LDDFQNLCCS

       370        380        390        400        410        420
ESFPLLKTMN RALGRSLPPI SEDCRRPPQP VTPQAPTLTT HSDGVDGNPH PTTSMTTSSS

       430        440        450        460        470        480
ATTWTERPST TTQQTTKTTW PTWTWTSQEP SKIASEDVTK LNETNKPSTT PSSTTPTSTI

       490        500        510        520        530        540
SCAPTTQLSH STTRKPIVDT SEANCTSGAY YSDPMNCGNY YRCVRGELKR EQCAPGLHWD
                          *            *      *          *
       550        560        570        580        590        600
ANRQLCDWPS AAKCQAETDS TSTTSSPLWS SSTKKPSTTT AKVTRPILTI TTTTTTIATT
     *        *
       610        620        630        640        650        660
KLTTSKPIVV EPTQKPQKPC EHGQYYPYPN SCTSFLVCVN GDLVSQQCGP GLNWNTDKNM
                    *             *     *          *
       670        680        690        700        710        720
CDWAFKSPCI NKPHKNALLV EKDTVPTTCI TGSYSGVPGD CTSYQACLWG RQEVFNCAPG
*       *                     *             *     *          *
       730        740        750        760        770
LHFNRETRIC DWPSRAKCAD RAAAASAGPR PPKPFLEQKL ISEEDLNSAV D

         *        *
```

Figure S3 - AsChtII-C2B3 deduced amino acid sequence. Conserved motifs in the glycoside hydrolase 18 family chitinases are underlined in red. The predicted sites for N-glycosylation are highlighted in dotted boxes. Six cysteine residues present in the CBM region are highlighted (black asterisk). The analysis of the sequence architecture of the sequence was developed using the SMART program (http://smart.embl-heidelberg.de/).

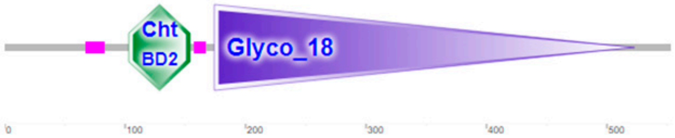


```
         10         20         30         40         50         60
EAEAEFDFKN RCGCEPSPLL RTMNRVLRNY PKGPLCPITE GTEVVIIDVD QSNIESTTVT

         70         80         90        100        110        120
ERPIYESTTS PTYIPPATIP TTTELDPDID DTIEIEASPL PPADCGGHLF ISHKSDCTKY
                                              *                *
        130        140        150        160        170        180
YLCNFGKISE QSCPSGLYWN EDRCDWPENT KCKTAESRSS LSPRLRLAKN VNEKKIVCYY
  *          *           *        *
        190        200        210        220        230        240
RIGLGRRASF GSFTPENIDG TLCTHIVYAF ATLEKETFLL NIDDSDNVYS SFLNKTAEIK

        250        260        270        280        290        300
KRNGLKVLLG LGGWNDSEDD KYSRLISSPQ STRKNFARYI VKIIEQYGFD GLNLDWEFPV
                                                                *
        310        320        330        340        350        360
CWXGDCSRGP EEDRENFVEF LKDLSEALTS RGLLLSIVVS ANKIVIDRGY THISLLTRYV

        370        380        390        400        410        420
NWIGVVAYDY HTGMGSETGH VAPLYHHPDD ISPFLNANFS VMYWINKGVP AEMIVLGIPT

        430        440        450        460        470        480
YGSSFTLSEK SLNRPSFHVK ADGPGEPGKF TKSAGFLAYH EICDNVKNDG WSVTKDSEGR

        490        500        510        520        530        540
MGPYASKRNQ WVSYDDVSNV AKKAELIKKL NLGGGMIWSL DLDDFRGLCG CGKYPLLTAL

        550
NNGLGRGNGH TTD
```

Figure S4 - AsChtII-C5B1 deduced amino acid sequence. Conserved motifs in the glycoside hydrolase 18 family chitinases are underlined in red. The predicted sites for N-glycosylation are highlighted in dotted boxes. Six cysteine residues present in the CDB region are highlighted (black asterisk). The analysis of the sequence architecture of a probable chitinase of leaf-cutting ants was developed using the SMART program (http://smart.embl-heidelberg.de/).

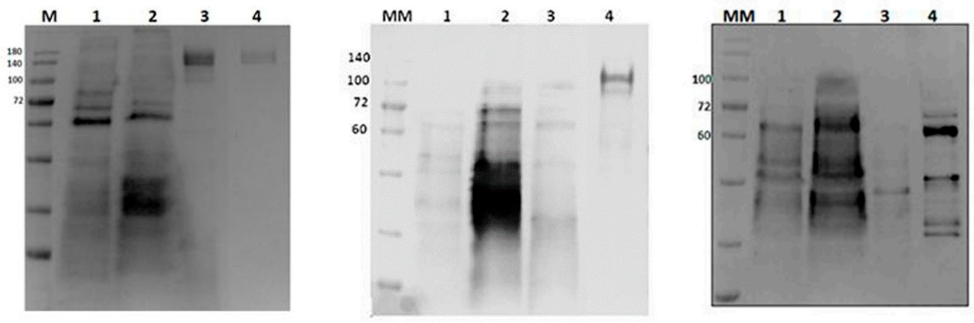

(A) **(B)** **(C)**

Figure S5 – SDS-PAGE of the proteins AsChtII-C2B3 (A), AsChtII-C3C4 (B), and AsChtII-C5B1 (C), **expressed in *P. pastoris*.**

In (A) and (B), M: molecular mass marker; 1: EE of the negative control (pPICZαA empty); 2 – EE applied to the Ni column; 3: solution eluted from the Ni column with 500 mM imidazole. 4: solution eluted of the Superdex 75 colum. In (C), M: molecular mass marker; 1: EE of the negative control (pPICZαA empty); 2 – EE applied to the Ni column; 3: solution that did not bond to the Ni column. 4: solution eluted of the Superdex 75 colum.

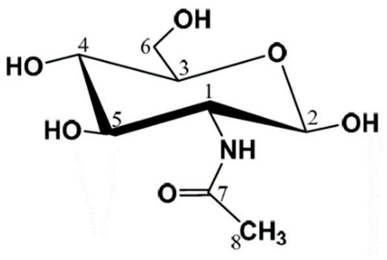

Figure S6– GlcNAc structure chemical

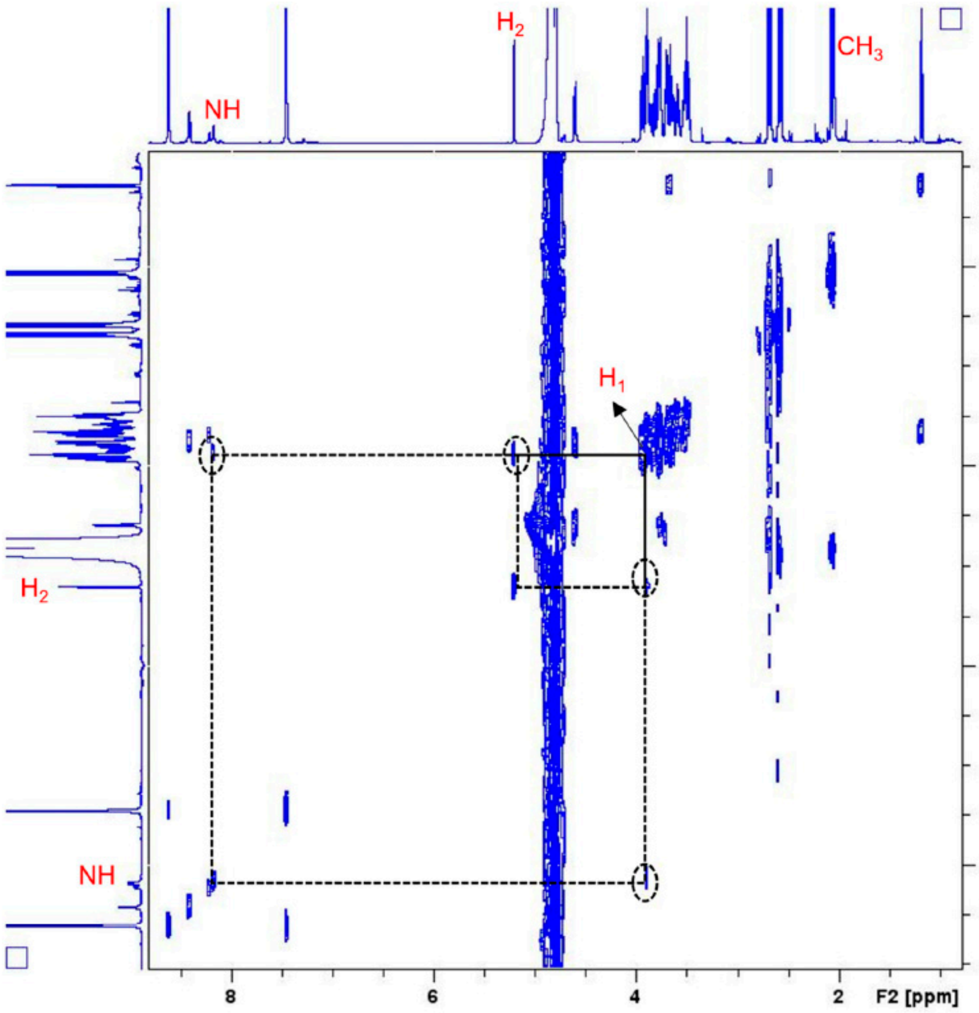

Figure S7 – COZY 1H-1H NMR spectrum for GlcNAc. Signals of the N-acetil-d-glucosamina produced are highlighted.
